# Intraindividual pain variability in chronic pain: A systematic review

**DOI:** 10.1177/17448069261439609

**Published:** 2026-04-09

**Authors:** Julie Klinke, Valentina Molinari, Karin Jensen

**Affiliations:** 1 Department of Clinical Neuroscience, Karolinska Institutet, Stockholm, Sweden

**Keywords:** Nociplastic pain, systematic review, pain variability, fibromyalgia, low back pain, temporomandibular disorder, osteoarthritis

## Abstract

**Objective:**

To present the first systematic review on the empirical evidence for variations in intraindividual pain intensity in patients with long-term pain conditions.

**Methods:**

The search was conducted on Medline and included prospective longitudinal non-interventional studies done on adult human subjects with chronic pain conditions linked to nociplastic pain mechanisms. Abstract screening and full-text review were performed by two independent reviewers. A third reviewer was consulted in case of disagreement.

**Results:**

Of the 1195 results, 13 studies fulfilled the eligibility criteria as determined by abstract screening and full-text review performed by two independent reviewers. Studies included patients with fibromyalgia, low back pain, temporomandibular disorder and, because of the growing evidence for central sensitization, osteoarthritis. Findings showed consistent evidence of intraindividual pain variability in patients with nociplastic pain, regardless of diagnosis. In several studies, it was possible to cluster patients based on degree of pain variability.

**Conclusions:**

Our findings provide evidence of intraindividual variability in pain intensity in patients with pain conditions that includes nociplastic pain mechanisms, regardless of precise diagnosis. Preliminary evidence suggests that degree of intraindividual pain variability may be associated with measures of clinical relevance, including mental health, fatigue, physical activity level, and drug and placebo response.

## Introduction

Pain is a universal human experience, but not all pain is created equal. Nociceptive pain arises from damage to non-neural tissue, and neuropathic pain results from lesions or diseases of the somatosensory nervous system. By contrast, nociplastic pain is characterized by altered nociception that can cause pain without clear evidence of tissue injury or nerve damage.^1^ Nociplastic pain is often present in conditions such as chronic low back pain (LBP), fibromyalgia (FM), and even osteoarthritis (OA), with nociplastic pain conditions affecting 2%–15% of the general population based on precise diagnosis.^2,3^ Across diagnoses, nociplastic pain is linked to reduced quality of life, loss of work capacity, and substantial healthcare costs, underscoring its individual and societal impact.^4^ Compared to other pain types, it is often refractory to conventional analgesics,^3,4^ highlighting the clinical importance and urgent need for more effective pain management strategies.

Patients with chronic pain shows substantial variability both inter- and intraindividually, with fluctuations in pain intensity and distribution over time.^3,5^ Patients often experience ‘good days’ and ‘bad days,’ reflecting marked day-to-day changes in symptom severity. Characterizing these temporal dynamics is critical for symptom management and for improving quality of life. Moreover, identifying the mechanisms underlying such fluctuations may provide insight into the origins and pathophysiology of nociplastic pain. However, most studies rely on cross-sectional group comparisons or single-timepoint assessments,^5^ approaches that fail to capture the inherently fluctuating nature of pain. Retrospective reports of pain intensity are also commonly used, though they are recognized as an unreliable measure of pain experience, especially for those with a high degree of intraindividual variability in their pain intensity.^6^

The aim of this systematic review is to synthesize existing evidence on temporal intraindividual variability of pain in patients with long-term pain conditions, with a specific focus on nociplastic pain. To our knowledge, this is the first systematic review of experimental studies examining intraindividual fluctuations in pain intensity over time in adults with chronic pain, independent of specific diagnosis.

### Methods

This study was conducted in accordance with the PRISMA guidelines. The study protocol was pre-registered at OSF (https://osf.io/pq72z). No changes were made after the preregistration.

#### Search strategy

The literature search was performed by VM using Medline. All research studies that fulfilled the following inclusion criteria were included: (a) human subjects, (b) adult subjects (>18 years), (c) chronic nociplastic pain conditions (>3 months), (d) longitudinal studies, (e) pain measures at least once per month, (f) intraindividual pain variability measures, (g) prospective measurements of pain. The following exclusion criteria applied: (a) animal models, (b) reviews, (c) meta-analyses, (d) pediatric subjects, (e) non-English articles, (f) mixed pain types, (g) acute pain (<3 months), (h) intermittent pain (e.g. migraine), (i) interventional studies without any prior stable pain measures before the intervention or intervention studies with no control group, (j) retrospective pain recall. The last search was conducted May 6, 2025. The search strategy was developed in Medline in collaboration with a librarian at the Biblioteca di Medicina Clinica at Bologna University, Italy. For each search concept Medical Subject Headings (MeSH-terms) and free text terms were identified. No language restriction was applied. Databases were searched from inception. Pediatric studies were excluded using available search blocks for Medline. De-duplication was done using Rayyan. The full search strategies for all databases are available in Supplemental Appendix.

After the search, titles and abstracts found in the literature search were screened for eligibility by two independent reviewers (JK, VM), using the Rayyan software for meta-analyses (Qatar Computing Research Institute). Secondly, two independent reviewers screened the eligibility of the full-texts articles (JK, VM). Any disagreements were resolved in discussion with a third reviewer (KJ). All data regarding sample size, characteristics of the study population, pain variability and conclusions were extracted by VM and randomly checked for correctness by JK. If there were any disagreement, a third reviewer was consulted (KJ). For more information about article inclusion and exclusion, see the Supplemental Appendix.

## Results

### Included studies

**Figure 1.**
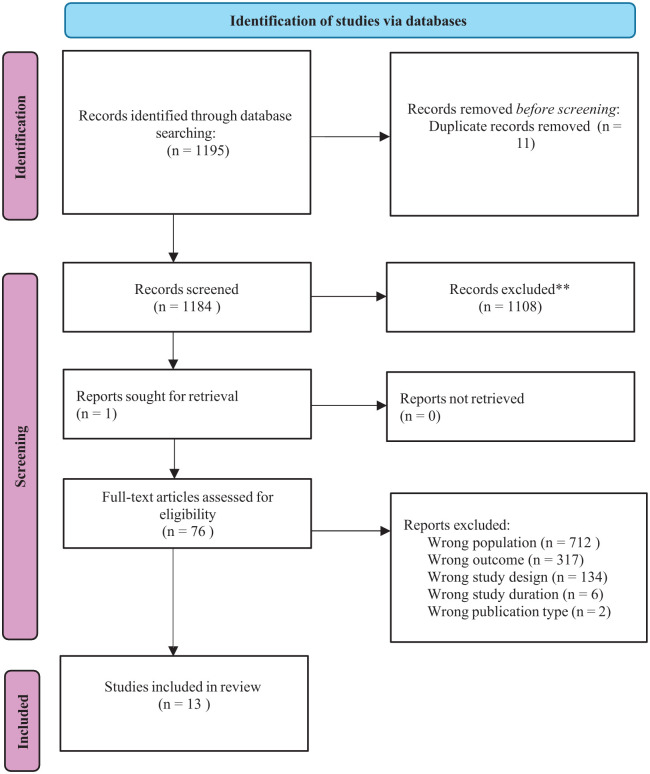
PRISMA flow diagram of literature search and selection.

The literature search initially identified 1195 titles and abstracts, of which 1184 were deemed potentially relevant. Following a full-text review of 76 articles, 63 were excluded for not meeting the predefined inclusion and exclusion criteria. Consequently, 13 studies met the eligibility criteria and were included in the review^7–19^ (see Figure 1 for the PRISMA flow diagram).

### Patient characteristics

The 13 included studies encompassed a total of 2831 participants. The majority of the participants were female (70.4%) with a mean age of 63.9 years (see Tables 1 and 2). The studies covered various nociplastic pain conditions, including patients with fibromyalgia (FM) (*k* = 3), osteoarthritis (OA) (*k* = 9), low back pain (LBP) (*k* = 2), and temporomandibular disorder (TMD) (*k* = 1).

**Table 1.** Information about study characteristics and main conclusions on intraindividual pain variability in nociplastic conditions.

Author, year	Country	Sample size (n)	Mean age	Sex	Pain condition	Total pain duration, years	Pain intensity measure	Pain variability assessment	Frequency of pain measure	Study duration	Study type	Type of outcome	Main conclusions
Bartley et al. (2018)	USA	256	48.5	F: 239 M: 16	FM	18.5	NRS (0–100) via phone pad with automated voice-response system	Hierarchical linear modelling of daily pain ratings	Once a day	90 days (min 2 weeks, max 90 days)	Obs	Primary outcome	FM participants showed significant intra- and interindividual variability in pain, fatigue, and mood. Three subgroups were identified: low, high, and mixed variability. Higher levels of pain were associated with greater fluctuation in pain, fatigue, and depression. Findings support the dynamic nature of FM pain.
Harris et al. (2005)	USA	125	47.05 (SD = 11.15)	F: 122 M: 3	FM	4.06 (SD = 4.16)	Gracely Box Scale (0–132, scaled down to 0–20) via electronic diary	Individual SD of sequential entries within 2-week time blocks calculated for each participant	Mean of 3.4 times per day	2 weeks	Obs/Int	Primary outcome	There were substantial interindividual differences in real-time pain reports among FM participants. Intraindividual pain variability was relatively stable over time. Pain variability appeared to be linked to drug responsiveness, moreover, participants with larger pain fluctuations responded better to placebo.
Macedo et al. (2014)	Canada	155	51.3	F: 94 M: 61	LBP	At least 3 months	NRS (0–10) via automated short message system, or by telephone for those who could not use text messages	Separate linear regression analysis used to predict pain as a function of log time; pain fluctuation identified if predicted pain deviated by ≥2 points from reported pain.	Once a month	1 year	Inter	Primary outcome	The study showed that only 21 (13.5%) of those participating in the trial had a fluctuating pain pattern over a 1-year period. These participants showed slightly greater baseline disability.
Van Grootel et al. (2005)	The Netherlands	133	31.4 (SD = 9.9)	F: 126 M: 7	TMD	1.9	VAS (100 mm) via paper diaries	Individual SD. Linear regression applied to daily VAS as function of day to examine sustained change in pain intensity. Repeated-measures ANOVA used to assess changes in mean daily VAS score between various week-days. Patients clustered based on the time of the day that maximum of mean VAS score occurred.	4 times a day	2 weeks	Obs/Int	Predictor	Although mean pain severity was relatively stable, the authors found high inter- and intraindividual day-to-day pain variability. Two main pain patterns were identified among participants with TMD: 79% reported higher pain later in the day, and 21% reported higher pain earlier in the day. These differences may reflect distinct processes influencing pain sensitivity and jaw muscle activation patterns.

FM: fibromyalgia; LBP: low back pain; NRS: numerical rating scale; SD: standard deviation; TMD: temporomandibular joint disorder; VAS: visual analogue scale.

**Table 2.** Information about study characteristics and main conclusions on intraindividual pain variability in osteoarthritis (OA).

Author, year	Country	Sample size (n)	Mean age	Sex	Pain condition	Total pain duration, years	Pain intensity measure	Pain variability assessment	Frequency of pain measure	Study duration	Study type	Type of outcome	Main conclusions
Allen et al. (2009)	USA	157	61.7 (SD = 10.6)	F: 81 M: 76	OA	/	VAS (0–100) via handheld computer diaries	Range of pain ratings (maximum pain rating minus minimum pain rating)	Every 2 hours	2 days	Obs	Primary outcome	There was substantial within-day variability in OA pain. Participants showed a decrease in pain intensity after the initial morning pain, followed by an increase and then a decline in the evening. Participants with hip and knee OA showed higher range in reported pain levels than patients with hand OA.
Allen et al. (2006)	USA	36	62.9 (SD = 10.4)	F: 11% M: 89%	OA	/	VAS (100 mm) via paper diaries	Median split (pain variability ≥1.5 or <1.5 on VAS) to cluster participants into high or low variability	Once a day	30 days	Obs	Secondary outcome	The study identified important interindividual differences in day-to-day variability with two subgroups: high vs low variability. Lower pain variability was linked to problem-focused coping, while higher variability was associated with emotion-focused coping. This was independent of mean pain levels.
de Koning et al. (2018)	Germany, Italy, the Netherlands, Spain, Sweden, UK	832	74.0	F: 548 M: 248	OA	/	NRS (0–10) via daily paper calendars	SD of each participant’s pain scores of all available days of the calendars; at least 7/14 days of at least 1 calendar had to be completed	Once a day	2 weeks at baseline and after 6 and 12–18 months (6 weeks)	Obs	Predictor	More severe pain was associated with a higher frequency of depression and anxiety. The study found significant day-to-day intraindividual variability in joint pain. Among older participants, greater fluctuations in pain (independent of average pain levels) were associated with less anxiety, depression, and other affective symptoms, while no significant association was found in younger participants.
Murphy et al. (2015)	USA	45	64.1 (SD = 10.0)	F: 24 M: 21	OA	/	NRS (0–10) via actiwatch; subjectively estimated pain flares recorded daily in logbook	Pain variability: SD of average activity counts per min Pain flare: defined during qualitative interviews; participants asked to use their own definitions when logging pain flares. Descriptive statistics used for each participants’ number of daily pain flares	8 times a day	7 days	Obs	Primary outcome	Most participants reported pain flares, often triggered by antecedents such as walking or climbing the stairs or prolonged sitting. Flares were often described as sharp and short in duration.
Nelson et al. (2024)	USA	252	53.5 (SD = 14.4)	F: 75.8% M: 24.2 %	OA (40.4%) FM LBP COPC (Chronic overlapping pain conditions)	12.8	NRS (0–10) via smartphone app	Pain ratings from the previous day subtracted from following day’s scores; differences converted to 0 (better) to 10 (worst) scale using min-max normalization method	Once a day	28 days	Obs	Secondary outcome	On average, participants reported stable pain levels, although some reported great levels of improvements and others worsening pain levels. Those with higher baseline pain, disability, catastrophizing, anxiety, and depression were more likely to underestimate improvements in pain intensity. Note that the authors asked participants to rate their experienced levels of change in pain as opposed to calculating pain variability based on measures of pain intensities, and that no distinction was made based on diagnosis.
Teirlinck et al. (2019)	The Netherlands	91	67(SD = 9.8)	F: 49 M: 42	OA	1.05	NRS (0–10) via diaries	Pain variability: SD of each participant’s pain scores Pain intermittency: Measure of Intermittent and Constant Osteoarthritis Pain questionnaire	Once a day	6 weeks	Inter	Primary outcome	Participants with higher intensity intermittent pain experienced more fluctuations compared to those with low intensity intermittent pain. Reliability between daily and retrospective measurements was lower in patients with higher intensity pain.
Timmermans et al. (2019)	Germany, UK, Italy, NL, Sweden, Spain	669	73.9 (SD = 4.9)	F: 70% M: 30%	OA	/	NRS (0–10) via daily paper calendars	SD of each participant’s pain scores from all available days	Once a day	2 weeks, repeated (calendar given at baseline and at 6-, 12-, and 18-month follow-up)	Obs	Predictor	Greater pain variability was associated with more physical activity in men, and with less (though not significantly) in women.
Treister et al. (2019)	Israel, USA	55	61.4 (SD = 10.2)	F: 35 M: 30	OA	At least 6 months	NRS (0–10) via actiwatch	SD of each participant’s pain scores collected during the baseline week, prior to randomization	Twice a day prompted by watch, with voluntary additional logging	1 week	Obs/Int	Secondary	Variability in both clinical and experimental pain ratings correlated with placebo responsiveness. Experimental pain variability predicted preferential placebo responsiveness.
Vivekanantham et al. (2023)	UK	25	65	F: 12 M: 13	OA	/	NRS (0–10) via cellular smartwatch	Difference between lunchtime and early-evening pain scores; scores were used for a *k*-means algorithm to cluster participants into sustained high levels, sustained low levels, and fluctuating levels of pain, as well as into a two-class (variance only) model of constant vs intermittent pain	Twice a day	90 days	Obs	Primary outcome	Among OA participants, there was little within-day but significant between-day pain variability. Participants could be categorized into three groups: intermittent pain vs constant pain with high or low levels. Fluctuating pain appeared to have a bigger impact on physical activity than stable levels of pain, whether high or low.

NRS: numerical rating scale; SD: standard deviation; VAS: visual analogue scale.

### Study characteristics

All included studies were published between 2005 and 2023. The duration of longitudinal pain assessments ranged from 2 days to 12 months, with most studies spanning 1 to 4 weeks. Eight studies employed observational designs, while five utilized baseline data from intervention trials. All but one study used either a numerical rating scale (NRS, 0–10) or a Visual Analogue Scale (VAS, 100 mm) for momentary pain measurements. The one study used a Gracely Box Scale, a logarithmically anchored 0–132-point scale that was scaled down to a 0–20 scale. Studies differed in how variability of intraindividual pain intensity was assessed. The most common method was calculation of individual participants’ standard deviations based on their pain scores over time. Other methods included pain range (maximum minus minimum pain), median splits, intraday differences, and linear regression models.

### Pain variability

#### FM

FM patients demonstrated marked intra- and interindividual variability in pain intensity, as well as in related symptoms such as fatigue and mood. Based on symptom fluctuations, three subgroups could be noted: individuals with low variability, high variability, and mixed variability across pain, fatigue, and depressive symptoms. Notably, higher average pain intensity levels were consistently associated with greater fluctuations in these domains, reinforcing the variable symptom profile of FM. The degree of intraindividual variability appeared to remain stable over time, suggesting that pain variability may be a trait-like characteristic for some FM patients. At the same time, substantial between-subject differences in real-time pain reports were observed, emphasizing the interindividual differences in FM symptoms. One study suggests that FM pain variability may be linked to treatment responsiveness, as patients with greater pain variability showed enhanced responsiveness to placebo interventions.

#### LBP

Only a subgroup of LBP patients (14%) exhibited fluctuating pain intensities when measured over the course of 1 year. This subgroup showed slightly higher baseline disability, suggesting a potential association between pain variability and functional impairment.

#### TMD

In patients with myogenous TMD, there was significant intra- and interindividual day-to-day variability in pain intensity. A daily pattern of increased pain later in the day was reported, suggesting underlying differences in pain sensitivity and muscle activation processes during the day.

#### OA

Studies including patients with OA found greater variability in pain intensity between days than within a single day, although notable within-day fluctuations were also observed in some cohorts. Morning pain was typically followed by a temporary decrease in pain intensity, then an increase most likely due to physical activity, and a decline again in the evening. Physical activity or strain such as prolonged sitting were reported as antecedents of a pain flare, described as short and stabbing pain. Patients could be broadly categorized into distinct pain profiles: intermittent pain, and constant pain at either high or low intensity levels. Those experiencing fluctuating or intermittent pain (particularly at higher intensities) reported more disruption to daily function compared to those with stable pain levels. Pain varied more in those with hip and knee OA than those with hand OA (Table 3).

**Table 3.** Details on possible confounders of the studies included in the review.

Author, year	Medications	Physical activity	Psychiatric comorbidities/overlapping medical conditions	Diagnosis	COVID period	Treated/untreated (surgery, injections, replacement)
Allen et al. (2009)	Patients were excluded if they had started a new analgesic or anti-inflammatory medication in the past 10 days; however, many participants had been on analgesic or anti-inflammatory drugs for longer. Pain medication use was documented.	Not assessed	Excluded patients with rheumatoid arthritis and significant vision or hearing problems	Patients identified based on the ICD 9th edition using electronic medical records or diagnosed by a physician based on radiographic evidence.	No	Not assessed
Allen et al. (2006)	Not assessed	Not assessed	Not assessed	Patients identified based on the ICD 9th edition using electronical medical records	No	Not assessed
Bartley et al. (2018)	Patients were tapered off analgesic and psychotropic medications; antidepressants <10 mg and trazodone <15 mg were allowed	Not assessed	Subjects excluded if they had any other significant medical condition	ACR 1990 criteria for FM (confirmed by a rheumatologist)	No	N/A
de Koning et al. (2018)	Analyses were adjusted for use of psychotropic medications and pain medications	Analyses were adjusted for physical activity	Analyses were adjusted for number of chronic diseases. Psychiatry comorbidities were not assessed.	Self-reported OA and clinical examination using ACR criteria	No	Not specified, but patients were included if they didn’t report joint replacement or surgery in the past 2 weeks
Harris et al. (2005)	Not assessed; patients were enrolled in a drug trial of milnacipran, so the stability of pain may have been influenced by treatment	Not assessed	Exclusion criteria were severe psychiatric illness (except major depressive disorder or generalized anxiety disorder) and/or other severe medical comorbidities	ACR 1990 criteria for FM (confirmed by a rheumatologist)	No	N/A
Macedo et al. (2014)	Use of analgesics was reported	Not assessed	Not assessed	Clinically diagnosed nonspecific LBP	No	N/A
Murphy et al. (2015)	Participants were excluded if they were on chronic opioids, antidepressants, or centrally acting knee pain medications	Measured	Participants were excluded if they had severe anemia, history of chronic severe kidney disease or hepatic impairment, or any allergies	Clinical OA diagnosis according to ACR criteria	No	Participants were excluded if they had had knee joint injections within the last 12 weeks and/or if they received concurrent treatment for knee pain
Nelson et al. (2024)	Not assessed	Not assessed	Participants were excluded if they had a present or past DSM-5 diagnosis of schizophrenia, delusional disorder, psychotic disorder, or dissociative disorder, and/or if they had any unstable systemic illness, condition requiring surgery, cancer, osteomyelitis, or bone disease	Diagnosis of chronic pain	Unclear	Not assessed
Teirlinck et al. (2019)	Use of pain medication in past 3 months was assessed	Not assessed	Patients were excluded if they had contraindications for exercise therapy because of comorbidity	Clinical OA diagnosis according to ACR criteria	No	Patients were excluded if they had had hip surgery or were on waiting list
Timmermans et al. (2019)	Medication use was assessed, referred to the use of analgesics, anti-inflammatory and/or anti-rheumatic drugs	Measured	Analyses were adjusted for number of chronic diseases. Psychiatry comorbidities were not assessed.	Self-reported OA and clinical examination using ACR criteria	No (data collection 2010–2011)	Not assessed
Treister et al. (2019)	Participants were not allowed to use analgesics during the study period	Not assessed	Patients excluded if they scored >12 on the Hospital Anxiety and Depression Scale and/or had any medical contraindications	Clinical OA diagnosis according to ACR criteria	No	Not assessed
Van Grootel et al. (2005)	Patients using psychoactive medication were excluded	Not assessed	Patients completed questionnaires including items rating psychological distress and life events	Diagnosed through clinical examination according to RDC criteria for TMD	No	Participants were excluded if they had received treatment within the last year
Vivekanantham et al. (2023)	Not controlled for	Measured (primary outcome)	Not controlled for	Self-reported symptomatic knee OA	No (data collection 2017–2018)	Not assessed

## Discussion

To date, most studies in patients with chronic pain have analyzed pain experience by measuring it at a single time point or by averaging across time. The present review aimed to fill this gap by describing temporal pain patterns of pain intensity. Here, we provide the first comprehensive overview of intraindividual pain fluctuations in chronic pain conditions, focusing on both nociplastic conditions as well as common conditions having a possible nociplastic component (e.g. OA). We included studies with prospective longitudinal pain measurements to examine intraindividual symptom patterns over different time periods. Such studies are more likely to accurately reflect fluctuations in patients’ lived pain experience as opposed to retrospective recall studies, which tend to be affected by momentary biases.^5^

Across chronic pain conditions, pain intensity shows substantial variability both within and between individuals, with fluctuations occurring across hours, days, and longer time spans. In OA and TMD, diurnal patterns were common, reflecting activity-related increases and evening declines. Patients with FM exhibited the highest degree of intra- and interindividual variability in pain intensity, often in parallel with changes in fatigue and mood, suggesting that temporal fluctuation of symptoms may be a hallmark of FM. In contrast, the data available in this review indicate that patients with LBP generally report stable pain intensities, with only a minority displaying fluctuating patterns associated with greater disability. Together, these findings highlight that intraindividual pain variability is not uniform across chronic conditions but may reflect distinct clinical phenotypes, with potential implications for prognosis, functional outcomes, and treatment responsiveness.

### Clustering based on variability

Several studies divided patients into clusters based on their pain profile, distinguishing groups with variable pain intensities from those with more constant pain. Notably, these profiles appeared to influence other important aspects of patients’ lives and clinical management, such as treatment response, physical activity levels, coping strategies, and psychological distress, although this effect may potentially be moderated by age and sex.^8,10,11,16,17,19^ Studying these clusters may shed light on individual differences between patients affected by the same nociplastic condition and could therefore result in better clinical evaluation and management. For example, one option could be to tailor the timing of patients’ use of medication based on their pain pattern throughout the day, or to encourage exercise at times where pain is particularly low.^7^

Some studies associated intraindividual pain variability with drug responsiveness. More specifically, individuals experiencing greater fluctuations in pain intensities were better placebo responders. In a previous randomized controlled double-blind pharmacological trial with SNRIs or placebo by our group,^1^ our group found a negative correlation between intraindividual pain variability and FM duration, meaning that patients’ perceived pain intensity tended to stabilize over time. This supports previous findings that the pain of patients that had had an FM diagnosis for longer were less affected by weather events than those with a more recent diagnosis.^20^ In one study,^1^ patients with a positive placebo response did not transition towards constant pain levels with longer FM duration. This suggests that a variable pain profile may be favorable for recruiting endogenous pain responses. Hence, studying pain variability and its mechanisms may enable the exploration of different, more tailored treatments, making it important for precision medicine. Of equal clinical importance is whether pain fluctuation profiles could be predictive of better or worse prognosis. Thus, studies investigating this would be of high scientific and clinical value.

### The inclusion of OA patients

Studies on OA were more abundant than those on other conditions. Specifically, 9 out of 13 studies included patients with OA, constituting 71% of the participants that were reported on in this review. It is important to note that unlike other pain conditions included in the review, which are commonly categorized as purely nociplastic or of mixed origin (e.g. CLBP), the underlying mechanism of OA pain is generally nociceptive. Despite this, the rationale of including OA is that patients with OA often develop a nociplastic component in the form of central sensitization or other alterations in nociceptive processing over time.^2^ This means that patients’ pain is likely to remain despite surgical interventions. Additionally, the search strategy identified only four studies whose participants’ pain conditions were clearly nociplastic in nature. Thus, we decided to include studies whose participants had OA under the assumption that it may still offer some insight into intraindividual variation in pain conditions with a nociplastic component. To avoid confusion, we made sure to clearly separate the results into two main sections, the first including studies on FM, CLBP, and TMD, and the second studies done on OA patients.

While fluctuating pain is considered a common characteristic of nociplastic pain disorders,^3^ this review has shown that there is surprisingly little longitudinal observational evidence that explicitly quantifies these fluctuations at the individual level. In this review, FM, TMD, and OA seemed to be characterized by intraindividual variations in pain intensity, while this was only a case for a subgroup of patients with CLBP. This indicates that intraindividual variability in pain may be a property of nociplastic pain but potentially be affected by as-of-yet undiscovered factors. Given the limited number of studies identified for this review, it remains a key question whether variability of pain intensity is comparable across conditions, and if so, whether it may imply shared mechanistic underpinnings across conditions. Future studies should aim to measure and report this phenomenon systematically.

#### Comparisons with nociceptive and neuropathic pain

Existing systematic reviews into other types of pain, including nociceptive and neuropathic pain as well as both experimental, acute, and long-term pain, have found evidence of a diurnal pattern of pain intensities. For example, acute and chronic inflammatory (nociceptive) pain conditions are at their most painful in the morning, as well as post-operative pain, fibromyalgia, trigeminal neuralgia, and migraines. In contrast, neuropathic pain, temporomandibular joint pain, and tension headaches generally increase in intensity later in the day, and cluster headache and labor pain appear strongest at night.^21–23^

One review found that the diurnal pattern of neuropathic pain is distinctly different from that of nociceptive pain, with neuropathic pain being consistently strongest in the afternoon, evening, and late at night.^22^ Potential causes of the circadian pattern of neuropathic pain may involve diurnal fluctuations of endogenous opioids, melatonin, substance P, and/or mood.^21,22^ The authors of the review additionally speculate that the diurnal pattern of neuropathic pain may be related to dysregulation of chloride ion channels in the dorsal horn of the spinal cord as a result of neuronal injury.^22^ Another systematic review reported greater diurnal variation of neuropathic pain among females and those with diabetic neuropathy as compared to males and those with postherpetic neuralgia.^21^ According to the review, no effect has been found of age, etiology, severity of short-form McGill pain questionnaire pain quality, and allodynia on pain intensity variability. However, it should be noted that this is based on the findings of only two studies. Circadian rhythmicity of pain intensity appears unaffected by analgesics.^21^

Thus, the existence of pain intensity variations across the day is well established, especially in conditions that are characterized by intermittent pain episodes such as migraine or tension headaches. However, most existing findings address group level trends when comparing the pain intensity patterns of different primary and secondary pain diagnoses. In contrast, few studies reflect individual differences in the degree of pain variability despite its potential relevance for disease classification and precision medicine.^5^

Several factors have been found to affect pain variability, both in nociplastic pain conditions and pain conditions with a clear underlying etiology. Like in our review, existing studies in patients with different types of pain disorders have found an association between greater intraindividual pain variability and higher scores on depression, pain catastrophizing, sleep disturbances, and placebo-response.^5,23–25^ However, research into which specific biopsychosocial factors may affect intraindividual pain variability, and to what degree pain variability may predict functioning, remains lacking.^5^ There is evidence for increased prevalence of mood disorders and disturbed sleep in patients with nociplastic pain disorders, such as fibromyalgia, compared to other pain populations.^5,26^ Given the preliminary evidence that this may be bidirectionally linked to greater pain intensity and variability, which in turn may predict treatment response, such research could hold important theoretical and clinical value – particularly for patients with nociplastic pain, where effective treatment options are limited. However, such potential causes and effects remain speculative at this stage and require further investigation across all pain conditions.

### Limitations

It is important to consider some limitations of the studies included in this review. Pain intensity itself was measured almost the exact same way across studies; however, consideration should be given to the heterogeneity of methods used to assess variability in pain intensity. The most common method was calculating individual standard deviations based on participants’ pain ratings or ranges of pain ratings, allowing for transparent insight into individuals’ pain scores. However, other studies clustered participants based on linear regression models, repeated-measure ANOVAs, in one case a median split and, in another case, based on logged numbers of self-assessed pain flares. Since most of the studies used standard deviations or pain ranges, we have summarized findings without discussing how they were calculated, but this diversity must be noted. Future research into intraindividual pain variability would greatly benefit from a standardized assessment tool.

About half of the studies did not exclude or adjust for psychiatric comorbidities. Conditions such as major depressive disorder or anxiety disorders are known to impact pain fluctuations, often resulting in greater pain variability.^24^ Second, about 50% of studies permitted patients to continue taking analgesic, anti-inflammatory, or psychoactive medications without reporting or adjusting for their use. This is likely to have affected pain ratings to some extent. Third, most studies (*k* = 9) did not measure physical activity levels and those that did were all focused on knee OA. Given the dual nociceptive-nociplastic component of OA,^2^ pain fluctuations may in part be related to different types and/or levels of activity as has been discussed in the reviewed studies, making it difficult to evaluate whether the reported pain fluctuations are in response to movement and if so, what this means for symptom management.

In addition to the limitations of the studies, this review itself presents some limitations that should be acknowledged. First, 70% out of the total number of participants were female, so variations in pain intensity may partly have been affected by the participants’ menstrual phase.^27^ However, nociplastic pain patients tend to be predominantly female across conditions,^3^ so the skewed sex division in the reviewed studies is likely to be representative of the relevant patient populations. Second, for this review, we had to exclude relevant articles even if they fulfilled the inclusion criteria because the authors, despite having collected the relevant data, did not report specific measures of intraindividual pain variability but only used the data for analyses. Since this review has found preliminary evidence that pain variability may be of interest for reasons related to both clinical treatment outcomes and potential mechanistic underpinnings, we suggest that future studies seek to report calculated intraindividual pain variability whenever possible.

Lastly, given the limited number of publications on purely nociplastic pain conditions that fulfilled our search criteria, we chose to include OA under the assumption that it would have a nociplastic pain component. However, since we could not determine the exact pain type in this patient group, it was not possible to determine whether the reported fluctuations were a result of nociplastic or nociceptive pain. The results from studies on OA may therefore not necessarily be applicable to other nociplastic conditions, and generalizations should thus be done with caution.

### Clinical implications

Nociplastic pain is notoriously difficult to treat.^3^ Describing and characterizing nociplastic pain behavior is necessary as a first step towards understanding its underlying mechanisms and what may cause, or prevent, fluctuations over time. Dividing patients into clusters based on their pain profile may be a way to identify optimal tailored pain treatments and their timing to obtain the best possible results. For this reason, future studies should also investigate whether the variability of pain intensities is more present in nociplastic pain as compared to nociceptive or neuropathic pain, and if so, whether this could meaningfully inform treatment strategy both for patients with nociplastic pain and patients with mixed pain.

## Conclusion

To our knowledge, this article is the first to show systematic evidence of intraindividual pain fluctuations as measured longitudinally in patients with chronic nociplastic pain. Preliminary evidence suggests that fluctuations may help predict clinical treatment outcomes and could be relevant for future research into the underlying mechanisms of nociplastic pain.

## Supplemental Material

sj-docx-1-mpx-10.1177_17448069261439609 – Supplemental material for Intraindividual pain variability in chronic pain: A systematic review Supplemental material, sj-docx-1-mpx-10.1177_17448069261439609 for Intraindividual pain variability in chronic pain: A systematic review by Julie Klinke, Valentina Molinari and Karin Jensen in Molecular Pain
