# Supplementary material for: Intraindividual pain variability in chronic pain: A systematic review
Source: Mol Pain. 2026 Apr 9;22:17448069261439609. doi: 10.1177/17448069261439609 (PMC13153499; doi:10.1177/17448069261439609)
Supplement: sj-docx-1-mpx-10.1177_17448069261439609 – Supplemental material for Intraindividual pain variability in chronic pain: A systematic review [file sj-docx-1-mpx-10.1177_17448069261439609.docx]

Documentation of search strategies

Date: May 2025

Topic/research question:

Name of researcher(s): Karin Jensen, Julie Klinke, Valentina Molinari

Databases:

1. Medline

Total number of hits:

- Before deduplication: 1195
- After deduplication: 1184

Text that can be used in the Methods-section:

Search strategy

A literature search was performed in the Medline database. The last search was conducted 2025-05-06.

The search strategy was developed in Medline in collaboration with a librarian at the Biblioteca di Medicina Clinica at Bologna University. For each search concept Medical Subject Headings (MeSH-terms) and free text terms were identified.

No language restriction was applied.

Databases were searched from inception.

Pediatric studies were excluded using available search blocks for Medline.

De-duplication was done in Rayyan.

The full search strategy is available in the appendix (1).

Studies included in review

(n = 13 )

**Identification of studies via databases**

**Screening**

Records screened

(n = 1184 )

Records excluded**

(n = 1108)

Reports sought for retrieval

(n = 1)

Reports not retrieved

(n = 0)

Full-text articles assessed for eligibility

(n = 76 )

Reports excluded:

Wrong population (n = 712 )

Wrong outcome (n = 317)

Wrong study design (n = 134)

Wrong study duration (n = 6)

Wrong publication type (n = 2)

**Included**

**Identification**

Records identified through database searching:

(n = 1195)

Records removed *before screening*:

Duplicate records removed (n = 11)

1. Medline

| Interface: PubMed  Date of Search: 2025-05-06  Number of hits: 1,195 | Search string:  ( "Nociplastic Pain"[MeSH Terms] OR "Nociplastic Pain"[Title/Abstract] OR "Chronic Pain"[MeSH Terms] OR "Chronic Pain"[Title/Abstract] OR "Fibromyalgia"[MeSH Terms] OR "Fibromyalgia"[Title/Abstract] OR "Irritable Bowel Syndrome"[MeSH Terms] OR "Irritable Bowel Syndrome"[Title/Abstract] OR "Complex Regional Pain Syndromes"[MeSH Terms] OR "complex regional syndrome"[Title/Abstract] OR "Low Back Pain"[MeSH Terms] OR "chronic low back pain"[Title/Abstract] OR "Osteoarthritis"[MeSH Terms] OR "Osteoarthritis"[Title/Abstract] OR "Neck Pain"[MeSH Terms] OR "chronic neck pain"[Title/Abstract] OR "Temporomandibular Joint Disorders"[MeSH Terms] OR "temporomandibular joint pain"[Title/Abstract] OR "Interstitial Cystitis"[MeSH Terms] OR "interstitial cystitis"[Title/Abstract] OR "Prostatitis"[MeSH Terms] OR "chronic pelvic pain syndrome"[Title/Abstract] OR "Musculoskeletal Pain"[MeSH Terms] OR "chronic musculoskeletal pain"[Title/Abstract] OR "Facial Pain"[MeSH Terms] OR "chronic orofacial pain"[Title/Abstract] ) AND ( "pain variability"[Title/Abstract] OR "pain fluctuation"[Title/Abstract] OR "pain instability"[Title/Abstract] OR "daily pain"[Title/Abstract] OR "day-to-day pain"[Title/Abstract] OR "temporal dynamics"[Title/Abstract] OR "pain patterns"[Title/Abstract] ) |
| --- | --- |
